# Risk-sensitive reproductive allocation: fitness consequences of body mass losses in two contrasting environments

**DOI:** 10.1002/ece3.1010

**Published:** 2014-03-03

**Authors:** Bård-Jørgen Bårdsen, Marius Warg Næss, Torkild Tveraa, Knut Langeland, Per Fauchald

**Affiliations:** 1 Norwegian Institute for Nature Research (NINA), Arctic Ecology Department, Fram CentreTromsø, NO-9296, Norway; 2 CICERO – Center for International Climate and Environmental Research, Fram CentreTromsø, NO-9296, Norway

**Keywords:** Evolution, individual optimization, individual quality, phenotypic plasticity, *Rangifer tarandus*, state dependence

## Abstract

For long-lived organisms, the fitness value of survival is greater than that of current reproduction. Asymmetric fitness rewards suggest that organisms inhabiting unpredictable environments should adopt a risk-sensitive life history, predicting that it is adaptive to allocate resources to increase their own body reserves at the expense of reproduction. We tested this using data from reindeer populations inhabiting contrasting environments and using winter body mass development as a proxy for the combined effect of winter severity and density dependence. Individuals in good and harsh environments responded similarly: Females who lost large amounts of winter body mass gained more body mass the coming summer compared with females losing less mass during winter. Additionally, females experienced a cost of reproduction: On average, barren females gained more body mass than lactating females. Winter body mass development positively affected both the females' reproductive success and offspring body mass. Finally, we discuss the relevance of our findings with respect to scenarios for future climate change.

## Introduction

It is well established that the problem of economic allocation of a limited budget is pertinent for studies of behavior (e.g., Stephens and Krebs 1986; Mace 1993; Næss and Bårdsen 2010; Næss et al. 2011). In biology, risk can be defined as unpredictable variation in behavioral outcomes that have consequences for an organism's fitness (Winterhalder et al. 1999; Winterhalder 2007). Risk sensitivity should be important whenever (1) the fitness function is nonlinear and (2) when one or more of the behavioral alternatives are associated by unpredictable outcomes (e.g., Stephens and Krebs 1986; Kuznar 2001, 2002; Kuznar and Frederick 2003). Risk sensitivity may be important for large mammalian herbivores because the relationship between environmental conditions and important population vital rates (e.g., age-specific survival and reproduction), and hence also fitness, is nonlinear (e.g., Henden et al. 2008; Koons et al. 2009). The combination of a harsh winter and low female autumn body reserves can, for example, have negative consequences for both reproductive success and adult survival (e.g., Clutton-Brock et al. 1996; Tveraa et al. 2003). Furthermore, benign winters do not represent bonanzas because neither survival nor reproduction is boosted above that of an average winter (Bårdsen 2009). This asymmetry between improved and worsened conditions represents a problem of risk because individuals cannot manipulate the probability of encountering a harsh winter, but may buffer the adverse consequences of meeting one by strategically reducing reproductive allocation to increase the likelihood of own survival (e.g., Bårdsen et al. 2011; Fig. 1 in Mautz 1978: provides a conceptual illustration).

**Figure 1:**
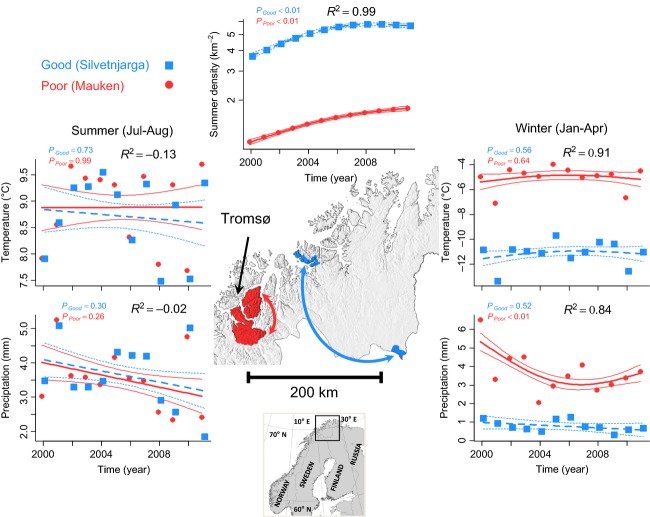
Map of the study area and information about population density (at the district level) as well as year-specific average values for winter and summer environmental conditions (based on gridded meteorological data) for the two study areas. Generalized additive models (GAMs) were used to assess potential temporal trends and the difference in average conditions for the good environment (adjusted *R*^2^ values are provided on the figures, but see Appendix S4 for other detailed GAM results).

It is widely accepted that populations are limited by both climate and population density (e.g., Turchin 1995; Sinclair and Pech 1996; Bonenfant et al. 2009). Increased density of reindeer *Rangifer tarandus* can, for example, have direct effects on individual body mass through increased competition for resources (Tveraa et al. 2007, 2013) and indirect effects through long-term negative effects on the pastures (Bråthen et al. 2007). A combination of short-and long-term effects creates ambiguous relationships between population density and individual-level responses. First, negative interactions between population density and late winter weather conditions are a source for potentially complex population dynamics (e.g., Coulson et al. 2001; Bårdsen et al. 2011). Second, individual buffering strategies, such as reduced reproductive allocation counteracting the impacts of both population density and climate, represent even further complications (see Bårdsen et al. 2008, 2009, 2010 for details). Nevertheless, it is well known that the combined effect of density dependence and climate will first manifest through body mass before any effects on survival and/or reproduction are detected (reviewed by e.g., Sæther 1997; Bonenfant et al. 2009).

Body mass is a state variable, or currency, that can be traded between reproduction and survival for many large herbivores, including reindeer (Bårdsen et al. 2011). Nevertheless, spring and autumn body mass represent two different states (e.g., Bårdsen et al. 2010), because the late winter season represents a bottleneck for survival (e.g., Coulson et al. 2001; Tveraa et al. 2003), whereas the summer represent a period of abundant forage (Bårdsen et al. 2010). Autumn body mass thus represents an insurance against winter starvation while during spring, when the risk of starvation is low, body mass is regulated down to a minimum threshold as the females seem to prioritize their newborn rather than increasing their own body reserves (Fauchald et al. 2004). Consequently, female reindeer have to make strategic choices on how to allocate resources between somatic growth and reproduction during summer. If too many resources are allocated to reproduction, autumn body reserves decrease (Bårdsen et al. 2008, 2009, 2010) resulting in subsequent reduction in the probability of surviving the following winter (Tveraa et al. 2003; Bårdsen et al. 2011). In essence, female reindeer pay the cost of their strategic resource allocation decisions made during summer the next winter (e.g., Bårdsen et al. 2008, 2010).

Body mass as an individual state has two major components, skeletal size and body composition (Festa-Bianchet 1998), where body composition represents both fat and protein reserves (e.g., Chan-McLeod et al. 1999; Monteith et al. 2013). For female *Rangifer tarandus*, fat and protein storages and body mass are to various degrees affected by external factors (e.g., seasonality and food shortage) and internal factors (e.g., reproductive status and age: Chan-McLeod et al. 1999; Barboza and Parker 2008; Thompson and Barboza 2013). As body mass is a strong predictor of both fat and protein (Gerhart et al. 1996; Thompson and Barboza 2013), we used body mass as a state variable representing body condition in this study.

We used data on semidomestic reindeer inhabiting contrasting environments during a time period when several overall system changes have occurred. Spring body mass has decreased over time, but female autumn body mass shows no apparent temporal trends (Bårdsen et al. 2010). During the same time period, population density has increased (Bårdsen et al. 2010; Næss et al. 2010, 2011; Næss and Bårdsen 2013), while large-scale climatic indices show that both winter and summer conditions have been fairly stable (Appendix S1). Female winter and summer body mass development appears to be mainly driven by density dependence and reproductive costs (see e.g., Bårdsen et al. 2010; Bårdsen and Tveraa 2012).

Previous studies have either provided experimental manipulation of winter feeding conditions (Bårdsen et al. 2008, 2009), analyzed observational data on absolute body mass (Bårdsen et al. 2010), or developed a simulation model (Bårdsen et al. 2011). This study assessed how winter body mass development affected female reproductive allocation using data from two contrasting environments because it is unknown whether body mass development affects individual traits similarly in poor vs. good environments. Consequently, this study used winter body mass development as a proxy for the combined effect of density and climate (but see e.g., Bårdsen and Tveraa 2012). As indicated above, large-scale climatic indices show no apparent trends (Appendix S1), but locally measured meteorological data (pixel size of 1 × 1 km containing day-specific values: Tveito et al. 2001 and references therein) can be used to divide our study area into a poor and a good area (Fig. 1). In the poor area, animals experience harsh, that is, poor and unpredictable, winter conditions with warm temperatures and more freeze–thaw events (e.g., Hansen et al. 2011) and more precipitation. In the good area, animals experience benign, that is, good and stable winter conditions with cold temperatures and less precipitation. Following the theory of risk sensitivity, we expect female reindeer that experience harsh winter conditions and a subsequent loss of body mass to (1) compensate for this loss by increasing the amount of resources allocated to own body mass during the following summer. Due to the cost of reproduction, we also predict summer body mass gains to be higher for barren than for lactating females (as successfully reproducing females are not capable of compensating for winter body mass losses to a similar extent as barren females). (2) We also expect offspring autumn body mass to be positively affected by maternal winter body mass development. Due to individual quality differences, measured by previous year's reproductive status (see Bårdsen et al. 2010; Bårdsen and Tveraa 2012), we also predict that females who were lactating the previous year to produce larger offspring than equally sized barren ones. (3) We expect reproductive success to be positively related to both female winter body mass development and past reproductive status. (4) We also expect animals exposed to harsh winter conditions on a long-term basis to be more risk averse (i.e., that they allocate fewer resources to reproduction and more to building own body reserves during summer).

## Materials and Methods

### Study populations and study area

This study was conducted on two populations of semidomestic reindeer in Norway (Appendix S1; Fig. 1): two herds from the same population in Finnmark and one herd in Troms (for details pertaining to the reindeer husbandry: see e.g., Fauchald et al. 2007; Tveraa et al. 2007; Næss and Bårdsen 2010; Næss et al. 2010; Ballesteros et al. 2012; Næss and Bårdsen 2013).

### Study protocol

From each herd, approximately 100 adult females (with unknown age >1.5 year) were individually marked when the study was initiated. Since then, we have followed the lineages produced by these individuals. Information about the data is published elsewhere (see Bårdsen et al. 2008, 2010; Bårdsen and Tveraa 2012 for details), but the data set contains the following variables:

*Winter body mass development* (WBMD) – The variable was created by subtracting previous autumn body mass [i.e., at year (*t*) − 1] from spring body mass (*t*).
*Summer body mass development* (SBMD) – The variable was created by subtracting autumn body mass from spring body mass (both measured within the same year).
*Offspring autumn body mass* – The variable measures offspring body mass in the autumn, the only season where we recorded offspring body mass.
*Reproductive status* (RS) – A variable that either acts as a binary variable (0 or 1, the response in the analyses of reproductive success;) or as a factor variable (the predictor in the analyses of body mass) depending on whether a female was barren or lactating.
*Previous reproductive status* (PRS) – This variable represents RS at *t *− 1.
*Time* – Year of the study, data were recorded 2002–2012 in Finnmark, and 2009–2011 in Troms.
*Id* – A factor variable giving each female a unique recognition (female identity used as categories or levels).
*Herd* – A factor variable with each population acting as levels.

### Statistical analyses – separated by area

Our hypotheses were tested by predicting (1) summer body mass development (a proxy for resources allocated to body reserves) as a function of winter body mass development (a proxy for climatic conditions and population density). In this analysis, we also tested to what degree barren females gained more body mass in summer compared with lactating ones. (2) Offspring body mass as a function of maternal winter body mass development. A positive effect in this analysis would indicate that females who experienced benign winter conditions produce larger offspring than those experiencing harsh conditions. (3) Reproductive success was modeled as a function of female winter body mass development.

We used linear mixed-effect models in the analyses of summer female body mass development and offspring body mass. Following Zuur et al. (2009), we selected and used one model for inference from a set of candidate models consisting of varying random and fixed effects (see Appendix S2 for details). As this study assessed how adult females allocate resources between reproduction and own body reserves, we excluded younger females from this analysis (i.e., <2 years). Generalized linear mixed-effect models were used for all analyses with a binary response variable (0 = “absent,” 1 = “present”; Appendix S2). In these analyses, we used a logit link function and a binomial distribution. We adopted the same model selection procedure as above. In order to reduce the effect of sexual maturation, we applied a stricter exclusion with respect to age in these analyses (as only females ≥3 years old were included in these analyses). Statistical analyses and plotting of results were carried out in *R* (R Development Core Team 2011), all tests were two-tailed, and the null hypothesis was rejected at an *α*-level of 0.05.

## Results

### Good environment (Finnmark)

**Figure 2:**
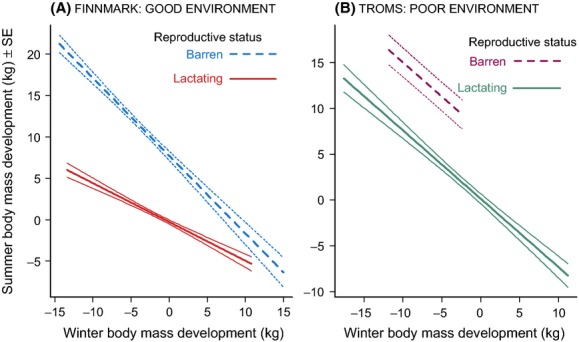
Summer body mass development as a function of winter body mass development and reproductive status for the good and poor environments, respectively. This shows the predictions and precision (±1 SE) from the model presented in Appendix S3:Table S3.1a and Table S3.2a. Please note that the range of values on the axes differs for the two areas.

Females that lost large amounts of body mass during winter gained more body mass during summer [due to a negative main effect of winter body mass development (WBMD): Appendix S3]. Summer body mass development was, however, on average ∼8 kg higher for barren compared with lactating females [main effect of reproductive status (RS)]. Additionally, the effect of winter body mass development was weaker for lactating compared with barren females (due to the positive interaction between RS and WBMD). Accordingly, barren females always gained more mass during summer compared with lactating ones. This difference decreased from ∼15 kg, for those with the highest loss of body mass during the previous winter, to ∼1 kg for those who gained the most body mass during the last winter (Fig. 2A).

**Figure 3:**
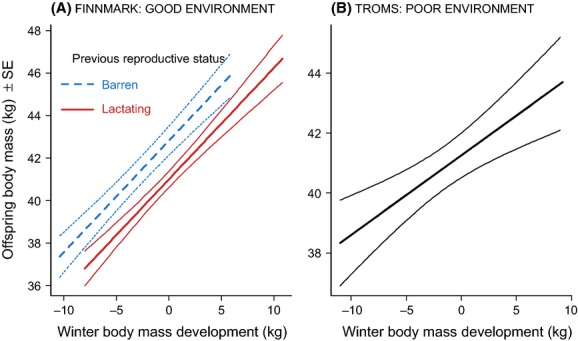
Offspring autumn body mass as a function of maternal winter body mass development and previous reproductive status for the good and poor environments, respectively. This shows the predictions and precision (±1 SE) from the model presented in Appendix S3:Table S3.1b and Table S3.2b. Please note that the range of values on the axes differs for the two areas.

**Figure 4:**
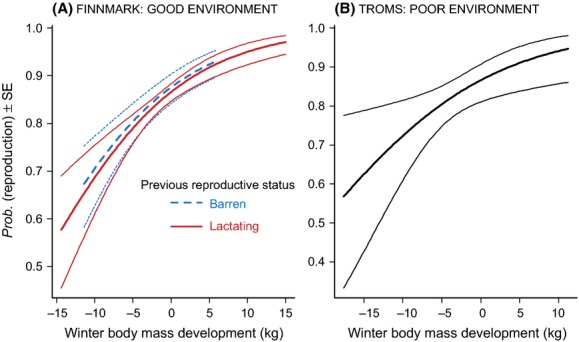
Reproductive success, that is, the probability of producing an offspring, as a function of maternal winter body mass development for the good and poor environments, respectively. This shows the predictions, on probability scale, and precision (±1 SE) from the model presented in Appendix S3:Table S3.3 and Table S3.4. Please note that the range of values on the axes differs for the two areas.

Offspring autumn body mass was positively related to the amount of maternal losses in winter body mass (positive main effect of WBMD) and negatively to previous reproductive status (negative main effect of PRS: Appendix S3). Consequently, females who were barren the previous year produced larger offspring compared with lactating females losing similar amounts of body mass (Fig. 3A). Females who lost large amounts of winter body mass experienced the lowest reproductive success [positive main effect of WBMD], but there was no difference between females who were barren or lactating the previous year [main effect of PRS (Fig 4A; Appendix S3)].

### Poor environment (Troms)

In the analysis of female summer body mass development, we documented almost similar effect sizes as in the good environment (negative main effects of both WBMD and RS: Appendix S3; Fig. 2B). Offspring autumn body mass was higher for females losing less body mass during the previous winter (positive main effect of WBMD), but the effect size was about half of that found for the good environment (Appendix S3; Fig. 3B). Females who lost large amounts of winter body mass experienced the lowest reproductive success even though this effect was not significant (Fig 4B; Appendix S3)].

## Discussion

Our results show that female reindeer compensate for large body mass losses during winter by increasing the gain in body mass the next summer. As the estimated effect sizes were similar (all the estimated effects were significantly negative), we also conclude that the same strategy is found in the two contrasting environments. We also documented both direct and lagged costs of reproduction as follows (1) females that reproduced were unable to buffer their winter losses to a similar extent as barren ones; and (2) barren females produced larger offspring the coming year compared with lactating ones (even though only in the good environment). Altogether, these findings indicate that female reindeer have adopted a risk-sensitive life history (see also Bårdsen et al. 2008, 2009, 2010; Bårdsen 2009), which has also recently been documented for elk *Cervus elaphus* and mule deer *Odocoileus hemionus* in North America (Morano et al. 2012; Monteith et al. 2013).

### Body mass development, body mass, and reproductive success

Females who lost large amounts of body mass during the last winter gained more mass during the subsequent summer. A cost of reproduction was apparent as barren females increased their body mass gain more during summer compared with lactating females. This supports findings from previous experiments which have shown that (1) barren females were larger in the autumn despite the fact that their early summer body mass was similar to lactating females (Tveraa et al. 2003; Fauchald et al. 2004; Bårdsen et al. 2009) and (2) after experiencing a harsh winter, females increased their own body mass gain the following summer at the expense of their offspring. This suggests that females reduce their reproductive allocation as a response to worsened winter conditions (Tveraa et al. 2003; Bårdsen et al. 2008). Reproductive allocation is therefore expected to affect autumn body mass to a larger extent than spring body mass, whereas spring body mass is expected to be more negatively affected by harsh environmental conditions and increased density than autumn body mass (Bårdsen et al. 2010; Bårdsen and Tveraa 2012).

When females gained body mass during winter, however, we documented an apparent reduced cost of reproduction as the difference between lactating and barren females was reduced. Even though we do not discriminate between negative density dependence, climate and a possible interaction (Bårdsen and Tveraa 2012) as possible mechanisms in our study, increased reindeer abundance is a more likely explanation compared with winter climatic conditions (Appendix S1, but see e.g., Bårdsen et al. 2010; Næss and Bårdsen 2010). An alternative explanation is, however, that the effects of density and climate must be understood in light of each other: At high density, harsh winters may have larger negative impacts on female reproductive allocation than at lower density. This may explain why several of the estimated effects are similar in the poor and good environments. Even though barren females increased their body mass gain during summer relative to lactating females (both environments), they still produced smaller offspring the subsequent year (good environment only). This suggests that individuals have different qualities (Weladji et al. 2008; Bårdsen et al. 2010), as this finding is the opposite of that expected from the cost of reproduction hypothesis (e.g., Williams 1966).

### Risk-sensitive reproductive strategies

Our findings support the hypothesis that female reindeer have adopted risk-sensitive reproductive strategies (e.g., Bårdsen et al. 2008, 2009). Risk sensitivity implies that individuals to some degree are either risk prone or risk averse. Female reindeer are supposed to be risk averse because (1) during the summer, they cannot predict climatic conditions the coming winter; and (2) harsh and benign winters have asymmetric fitness consequences. In other words, female reindeer cannot assume that a coming winter will be benign, because the cost of preparing for a benign winter and meeting a harsh one is dramatically higher than the benefit of preparing for a benign winter and actually get one. Consequently, female reindeer optimized their allocation between reproduction and body reserves according to expected winter conditions (a conclusion that is supported by comparing the results presented in Figs 4).

Risk sensitivity may also be relevant for other large temperate herbivores: Many herbivores accumulate fat during summer and experience reduced survival the coming winter if they lose body reserves during summer (e.g., Soay sheep *Ovis aries* and red deer *Cervus elaphus*: Clutton-Brock et al. 1997). Allocating resources to reproduction thus seems to represent a “lost opportunity for gain.” Moreover, the cost of reproduction is paid both directly and indirectly. Directly by the fact that reproducing females experiences lowered summer body mass gain, and indirectly because reproducing females who lost large amounts of body reserves during winter also produce the smallest offspring (e.g., Bårdsen and Tveraa 2012). Consequently, female reproductive allocation must be understood in the context of potential reproductive rewards. If, for example, the coming winter will be harsh, females with the smallest offspring will experience large indirect and direct costs: Their offspring will almost certainly die while at the same time they are paying a cost of lactation (during severe winters even smaller adults will experience reduced survival: Tveraa et al. 2003).

Many of the assumptions for risk-sensitive reproductive allocation are, at least partly, fulfilled for many long-lived organisms. Long-lived organisms experience a temporally varying cost of reproduction, they build body reserves during periods of favorable environmental conditions, and they use these reserves as a buffer against unpredictable environmental variability during periods of nonfavorable conditions [e.g., humans (e.g., Bronson 1995; Lummaa and Clutton-Brock 2002), large herbivores in general (Sæther 1997; Gaillard and Yoccoz 2003), birds (Parker and Holm 1990; Hanssen et al. 2005), fish (van den Berghe 1992; Hutchings 1994; Klemetsen et al. 2003), and reptiles (Shine 2005; Radder 2006)].

## Conclusions

The ability for individuals to buffer negative climatic effects by adopting a risk-sensitive reproductive strategy has important consequences for how future climate change may affect biological populations (Bårdsen et al. 2011). In fact, many climatic responses found in the literature on long-lived organisms can be interpreted as signatures of risk-sensitive reproductive allocation strategies as follows (1) Harsh winter conditions are expected to increase mortality, notably for small individuals (e.g., Albon et al. 1983; Clutton-Brock et al. 1996); (2) harsh environmental conditions have been found to delay the onset of reproduction and lower reproductive effort (e.g., Sæther et al. 1996; Sand 1996); and (3) under these conditions, only larger females are expected to reproduce (e.g., Albon et al. 1983; Festa-Bianchet et al. 1998). Understanding the mechanisms in which climate affects the evolution of life-history strategies will be vital in predicting both the demography and population viability for long-lived organisms in response to future climate change (see e.g., Simard et al. 2008; Monteith et al. 2009 for similar conclusions).

## Supporting Information

Appendix S1 Temporal trends in climate and reindeer abundance.

Appendix S2 Model selection.

Appendix S3 Parameter estimates.

Appendix S4 Using GAMs to assess temporal trends.
