# Supplementary material for: Risk-sensitive reproductive allocation: fitness consequences of body mass losses in two contrasting environments
Source: Ecol Evol. 2014 Mar 3;4(7):1030–8. doi: 10.1002/ece3.1010 (PMC3997319; doi:10.1002/ece3.1010)
Supplement: Appendix S1 — Temporal trends in climate and reindeer abundance. [file ece30004-1030-sd1.pdf]

S1: TEMPORAL TRENDS IN CLIMATE AND REINDEER ABUNDANCE

The number of reindeer in Norway, especially in Finnmark, has been characterized by considerable temporal variation through the last century. From early 1900 there was a decreasing trend that reached a minimum around World War II. After the war, the numbers have been fluctuating in an upward trend that peaked in the early 1990s, decreased until 2000/2001 and then again has increased in recent years. In 2010 the number of reindeer again reached a historical high level (Tømmervik and Riseth 2011). This temporal increase is evident at the national level (Næss et al. 2011) as well as for many districts (Tveraa et al. 2007, Bårdsen et al. 2010) and husbandry units in Finnmark (Næss and Bårdsen 2010). From 2002, when our demographic studies on reindeer in Finnmark started and onwards, herd size for individual owners has increased both in Finnmark (Fig. S1.1a) and in Troms (Fig. S1.1b). During the same period, large-scale climatic indices, common to both areas, of winter and summer conditions have been fairly stable (Fig. S1.1c): here measured using the Arctic Oscillation Index (see also Aanes et al. 2002). In Finnmark, spring body mass has, however, decreased while autumn body mass has not showed any significant temporal trends (Fig. S1.1d). In Troms there are data from too few years to estimate any temporal trends in any population-level averages (Fig. S1.1e).

LITERATURE CITED

Aanes, R., B.-E. Sæther, F. M. Smith, E. J. Cooper, P. A. Wookey, and N. A. Øritsland. 2002. The Arctic Oscillation predicts effects of climate change in two trophic levels in a high-arctic ecosystem. Ecology Letters 5:445-453.

Bårdsen, B.-J., T. Tveraa, P. Fauchald, and K. Langeland. 2010. Observational evidence of a risk sensitive reproductive allocation in a long-lived mammal. Oecologia 162:627-639.

Næss, M. W. and B.-J. Bårdsen. 2010. Environmental stochasticity and long-term livestock viability - herd-accumulation as a risk reducing strategy. Human Ecology 38:3-17.

Næss, M. W., B.-J. Bårdsen, E. Pedersen, and T. Tveraa. 2011. Pastoral herding strategies and governmental management objectives: predation compensation as a risk buffering strategy in the Saami reindeer husbandry. Human Ecology 39:489-508.

Tveraa, T., P. Fauchald, N. G. Yoccoz, R. A. Ims, R. Aanes, and K. A. Høgda. 2007. What regulate and limit reindeer populations in Norway? Oikos 116:706-715.

Tømmervik, H. and J. Å. Riseth. 2011. Historiske tamreintall i Norge fra 1800-tallet fram til i dag. NINA Rapport 672. 36 pp. (in Norwegian)

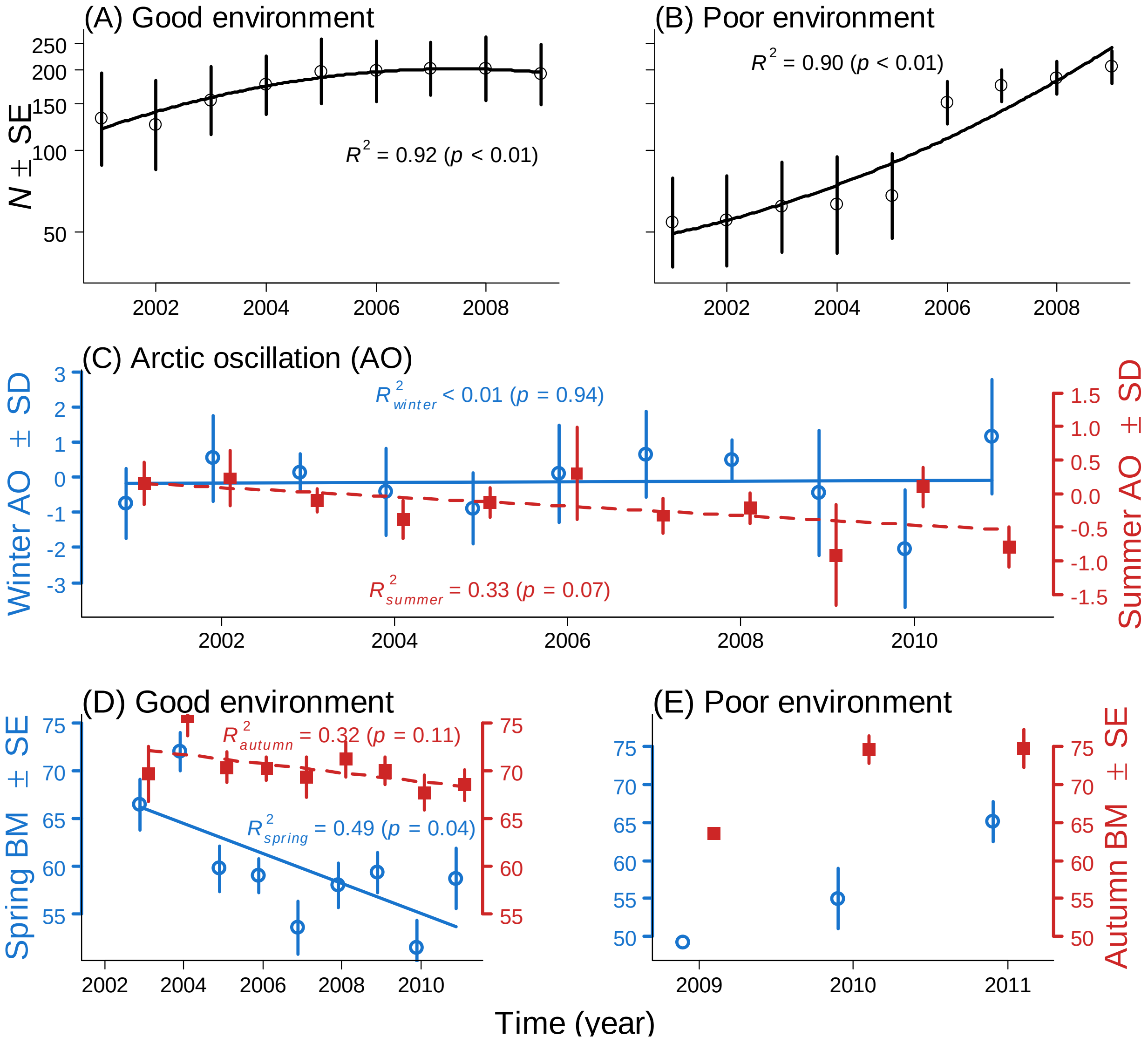

Fig. S1.1. Temporal trends in average: (1) number of female reindeer per owner; (b) summer and winter climatic conditions [Winter Arctic Oscillation (AO[1]) is the December (previous year)-April monthly average, whereas summer AO is the June-August monthly average]; and (c) spring and autumn body mass (for barren females only).

[1] http://www.cpc.ncep.noaa.gov/products/precip/CWlink/daily_ao_index/monthly.ao.index.b50.current.ascii.table.
