# Supplementary material for: Risk-sensitive reproductive allocation: fitness consequences of body mass losses in two contrasting environments
Source: Ecol Evol. 2014 Mar 3;4(7):1030–8. doi: 10.1002/ece3.1010 (PMC3997319; doi:10.1002/ece3.1010)
Supplement: Appendix S2 — Model selection. [file ece30004-1030-sd2.pdf]

S2: MODEL SELECTION

BODY MASS AND BODY MASS DEVELOPMENT

We selected a single model through a number of different steps (following Zuur et al. 2009). First, we selected the most parsimonious random effect structure conditional on the 'full fixed effect model'. We kept the most complex fixed effect structure, reflecting our most complex *a priori* biological hypothesis, constant in this step in the selection process: winter body mass development, reproductive status (RS; analysis 1), or previous reproductive status (PRS; analysis 2), and the two-way interaction between them. All models were fitted using a restricted maximum likelihood and we tested three different random effects structures: (1) random slopes and intercepts per individual; (2) random intercepts fitted per individual; and (3) no random effects. Second, we selected the most parsimonious fixed effect structure conditional on the selected random effect structure from the first step. We tested three different fixed effects structures (all models were fitted using maximum likelihoods): (1) an interaction model represented by the full model above; (2) an additive model containing the main effects of winter body mass development and RS or PRS: and (3) an additive model containing only winter body mass development. Third, the selected model from the set of candidate models was then re-fitted using a restricted maximum likelihood.

Selecting the models used for inference in each of the steps described above was performed using *Akaike's Information Criterion* (AIC) (e.g. Buckland et al. 1997, Anderson et al. 2000, Burnham and Anderson 2002). For each of the steps above we rescaled and ranked models relative to the model with the lowest AIC value (Δ_i denotes this difference for model i), and we selected the simplest model with a $\Delta_i \leq 2$. Additionally, we used standard modelling diagnostics plots in order to assess if the selected models fulfilled the underlying assumptions for these models (e.g. Zuur et al. 2010). Linear mixed-effect models were applied using the lme function in the nlme package (Pinheiro et al. 2012), whereas candidate models without random effects were fitted using the gls function in the nlme package (Table S2.1).

Plotting of predictions, including precision, was performed using the predictSE.lme function in the package AICcmodavg (Mazerolle 2013) for the mixed-effects models and the predict.lm for the models fitted using the lm function (selected models without random effects were re-fitted using the lm function).

REPRODUCTIVE SUCCESS

Generalized linear mixed-effect models (glmer), using the lme4 package (Bates et al. 2012), were used in all analyses where reproductive success was a response (binary variable: 0 = barren or 1 = lactating, Table S2.2). We adopted the same model selection philosophy in these analyses as described above, except that all models were fitted using Laplacian approximation (Bolker et al. 2009). In a previous version of the lme4-library, we were able to fit a dummy variable consisting of just a constant as follows: '(…) + 1|dummy'. This made it possible to compare models with and without random effects directly using the same likelihoods, and the AIC values presented in Table S2.1 is based on this approach. Please note that in the current version of the lme4 package this is no longer possible, i.e. as of now we are unable to extract and compare AIC values for models with and without random effects. As model selection using AIC is no longer an option, below we report the actual parameter estimates for both the selected model and the other candidate models.

In the analysis of female reproductive success for the good area, the parameter estimates for WBMD changed from 0.108 in the selected model (Table S3.3 in Supplement S3) to 0.088 (for

both the model with random intercept only and without random effects). For PRS, the parameter estimate changed from -0.088 in the selected model (Table S3.3) to -0.014 (for both the model with random intercept only and without random effects). In other words, the direction of the relationships did not change between models: selecting one model over the others had no impact on the conclusions drawn.

In the analysis of female reproductive success for the poor area, the parameter estimates for WBMD (Table S3.4) changed from 0.091 in the selected model and the model with random intercepts only to 0.186 in the model with random intercept and slope. The most complex mixed model resulted in an increased effect size, whereas the other mixed effect model resulted in a similar effect size as estimated in the selected model. The selected model is thus conservative since it leads to a parameter estimate that are either weaker or similar to the other candidate models, but again the direction of the relationships did not change depending on the selected model.

Plotting of predictions, including precision, were performed using the predictSE.mer function in the package AICcmodavg (Mazerolle 2013) for the mixed-effects models and the predict.glm for the models fitted using the glm-function.

LITERATURE CITED

Anderson, D. R., K. P. Burnham, and W. L. Thompson. 2000. Null hypothesis testing: problems, prevalence, and an alternative. Journal of Wildlife Management 64:912-923.

Bates, D., M. Maechler, and B. Bolker. 2012. lme4: linear mixed-effects models using S4 classes. R package version 1.0-5.

Bolker, B. M., M. E. Brooks, C. J. Clark, S. W. Geange, J. R. Poulsen, M. H. H. Stevens, and J. S. S. White. 2009. Generalized linear mixed models: a practical guide for ecology and evolution. Trends in Ecology & Evolution 24:127-135.

Buckland, S. T., K. P. Burnham, and N. H. Augustin. 1997. Model selection: an integral part of inference. Biometrics 53:603-618.

Burnham, K. P. and D. R. Anderson. 2002. Model selection and multimodel inference: a practical information-theoretic approach. Second edition. Springer, Inc., New York, USA.

Mazerolle, M. J. 2013. AICcmodavg: Model selection and multimodel inference based on (Q)AIC(c). R package version 1.35.

Pinheiro, J. C., D. M. Bates, S. DebRoy, S. Deepayan, and R. D. C. Team. 2012. nlme: linear and nonlinear mixed effects model. R package version 3.1-111.

Zuur, A. F., E. N. Ieno, and C. S. Elphick. 2010. A protocol for data exploration to avoid common statistical problems. Methods in Ecology and Evolution 1:3-14.

Zuur, A. F., E. N. Ieno, N. J. Walker, A. Saveliev, A., and G. M. Smith. 2009. Mixed effects models and extensions in ecology with R. Springer, USA.

Table S2.1. The relative evidence for each candidate model (i), in the assessment of random effects, based on differences in AIC values (Δ_i) for the statistical analyses of the population in the poor environment (Troms) and the good environment (Finnmark). The model **underlined in bold** was selected and used for inference. The 'full model' with respect to the fixed effects is similar to model 1 in Table S2.2. Female id (Id) represents the grouping effect, and the intercept means that the constant varied across groups whereas slope refers to a varying coefficient for winter body mass development (WBMD) across groups.

	Intercept and slope[a]		Intercept[b]		No rand. effects[c]	
Response	Poor	Good	Poor	Good	Poor	Good
Summer body mass development[d]	5.981	5.308	2.000	2.000	**0**	**0**
Offspring body mass[e]	6.000	0.895	2.000	**0**	**0**	**1.187**
Reproductive success[f]	2.962	**0**	0	2.995	**≤0.001**	2.995

[a]The syntax for the random effects were as follows: 'random = ~WBMD|id' (for models fitted via a call to the lme function); and '(…) + WBMD|id' (for models fitted via a call to the glmer function).
[b]The syntax for the random effects were as follows: 'random = ~1|id' (for models fitted via a call to the lme function); and '(…) + 1|id' (for models fitted via a call to the glmer function).
[c]For the responses using lme (body mass) we used the gls function (using method = "REML").
[d]The sample size were 599 in the analyses of populations in Finnmark [degrees of freedom (df) = 8, 6 and 5 for the different models (going from left to right)] and 50 for the analyses of the herd in Troms (df = 5, 4 and 3).
[e]The sample size were 599 in the analyses of populations in Finnmark (df = 8, 6 and 5) and 31 for the analyses of the herd in Troms (df = 6, 4 and 3).
[f]The sample size were 599 in the analyses of populations in Finnmark (df = 5,5 and 2) and 70 for the analyses of the herd in Troms (df = 3, 2 and 3).

Table S2.2. The relative evidence for each candidate model (*i*), in the assessment of different fixed effects, based on differences in AIC values (Δ_i) for statistical analyses of the population in the good environment (Finnmark).

i	Winter body mass development (WBMD)[a]	Reprodcutive success (RS)/Previous reproductive success PRS)[b]	RS/PRS × WBMD	Summer body mass development		Offspring body mass		Reproductive success	
				df[c]	Δ_i	df[c]	Δ_i	df[c]	Δ_i
1	x	x	x	**5**	**0**	5	0	5	3.780
2	x	x		4	14.817	**4**	**1.776**	**4**	**1.910**
3	x			3	288.249	3	5.270	4	0

[a]This predictor was kept in all models based on our *a priori* expectations.
[b]Reproductive success (RS) was used in the first analysis whereas previous reproductive success (PRS) were used in the other analyses (see main text for details).
[c]df denotes the number of parameters, whereas the number of observations is provided in Table S2.1.

Table S2.3. The relative evidence for each candidate model (*i*), in the assessment of different fixed effects, based on differences in AIC values (Δ_i) for statistical analyses of the population in the poor environment (Troms).

i	Winter body mass development (WBMD)[a]	Reprodcutive success (RS)/Previous reproductive success PRS)[b]	RS/PRS × WBMD	Summer body mass development		Offspring body mass[d]		Reproductive success[d]	
				df[c]	Δ_i	df[c]	Δ_i	df[c]	Δ_i
1	x	x	x	5	1.993	-	-	-	-
2	x	x		**4**	**0**	-	-	-	-
3	x			3	15.233	-	-	-	-

[a]This predictor was kept in all models based on our *a priori* expectations.
[b]Reproductive success (RS) was used in the first analysis whereas previous reproductive success (PRS) were used in the other analyses (see main text for details).
[c]df denotes the number of parameters, whereas the number of observations is provided in Table S2.1.
[d]Data on PRS was too scarce to include as a predictor in this analysis. Consequently, the only predictor included as a fixed effect in this analysis was winter body mass development (WBMD).
