# Supplementary material for: Risk-sensitive reproductive allocation: fitness consequences of body mass losses in two contrasting environments
Source: Ecol Evol. 2014 Mar 3;4(7):1030–8. doi: 10.1002/ece3.1010 (PMC3997319; doi:10.1002/ece3.1010)
Supplement: Appendix S3 — Parameter estimates. [file ece30004-1030-sd3.pdf]

S3: PARAMETER ESTIMATES

Estimated parameters for the models selected and used for inference (see main text and Supplement S3 for details) in the analyses of body mass and body mass development (Tables S3.1: good area & Table S3.2: poor area) and in the analysis of reproductive success (Table S3.3: good area & Table S3.4: poor area).

Table S3.1. Estimates from linear models relating (a) summer body mass development and (b) offspring autumn body mass to winter body mass development and covariates (for the good area: see main text and Supplement S3 for details).

Parameter	Estimate	SE	*t*	*P*
(a) Summer body mass development (kg)[a]				
Intercept	7.707	0.551	14	<0.001
Winter body mass development (WBMD)	-0.938	0.092	-10	<0.001
Reproductive success (RS) [lactating]	-7.994	0.606	-13	<0.001
RS [lactating] × WBMD	0.470	0.114	4	<0.001
(F = 226.1; df = 3,595; P < 0.01; R^2 = 0.53)				
(b) Offspring body mass (kg)[a]				
Intercept	42.825	0.688	62.277	<0.001
Winter body mass development (WBMD)	0.526	0.095	5.561	<0.001
Previous reproductive success (PRS) [lactating]	-1.819	0.777	-2.342	0.020
(F = 15.8; df = 2,173; P < 0.01; R^2 = 0.15)				

[a]Since the selected model did not contain any random effects the parameter estimates reported here are from a regular lm-fitted model.

Table S3.2. Estimates from linear models relating (a) summer body mass development and (b) offspring autumn body mass to winter body mass development and covariates (for the poor area: see main text and Supplement S3 for details).

Parameter	Estimate	SE	*t*	*P*
(a) Summer body mass development (kg)[a]				
Intercept	7.563	1.659	4.559	<0.001
Winter body mass development (WBMD)	-0.747	0.090	-8.322	<0.001
Reproductive success (RS) [lactating]	-7.423	1.688	-4.398	<0.001
(F = 56.4; df = 2,47; P < 0.01; R^2 = 0.71)				
(b) Offspring body mass (kg)[a]				
Intercept	41.258	0.745	55.349	<0.001
Winter body mass development (WBMD)	0.266	0.131	2.034	0.051
(F = 4.1; df = 1,29; P < 0.05; R^2 = 0.13)				

[a]Since the selected model did not contain any random effects the parameter estimates reported here are from a regular lm-fitted model.

Table S3.3. Estimates from generalized linear mixed-effect models relating female reproductive success to winter body mass development (for the good area: see main text and Supplement S3 for details).

Parameter	Estimate	SE	*z*	*P*
(a) reprodcutice success (logit-scale)				
Fixed effects				
Intercept	1.952	0.281	6.900	<0.001
Winter body mass development (WBMD)	0.108	0.037	2.900	0.004
Previous reproductive success (PRS) [lactating]	-0.088	0.293	-0.300	0.763
Random effects				
Intercept (among-individual SD)	0.184		$n_{\text{Observation}}$ = 532	
WBMD (among-individual SD)	0.037		n_{Groups} = 199	

Table S3.4. Estimates from generalized linear models relating female reproductive success to winter body mass development (for the poor area: see main text and Supplement S3 for details).

Parameter	Estimate	SE	*z*	*P*
(a) reprodcutice success (logit-scale)[a]				
Intercept	1.868	0.415	4.499	<0.001
Winter body mass development (WBMD)	0.091	0.067	1.357	0.175
(*df* = 68; Residual deviance = 62.14)				

[a]Since the selected model did not contain any random effects the parameter estimates reported here are from a regular glm-fitted model.
