# Supplementary material for: Risk-sensitive reproductive allocation: fitness consequences of body mass losses in two contrasting environments
Source: Ecol Evol. 2014 Mar 3;4(7):1030–8. doi: 10.1002/ece3.1010 (PMC3997319; doi:10.1002/ece3.1010)
Supplement: Appendix S4 — Using GAMs to assess temporal trends. [file ece30004-1030-sd4.pdf]

S4: USING GAMs TO ASSESS TEMPORAL TRENDS

The relationships between year-specific averages for precipitation, summer and winter temperatures and density for the two study populations (Fig. 1 in the main text) was modelled with generalized additive models (GAM) using the mgcv library (Wood 2012) in R. Thin plate regression splines were used to model potential non-linear effects of year. The degree of complexity for each of the smooth terms in the GAMs were limited by 'k', set to 4 in the present study (Wood 2006). The effective degrees of freedom (edf) measure the degree of smoothness between a given predictor and the response: an edf of 1 represents a linear relationship, an edf of 2 represents a second-order polynomial, and a value in between (i.e. an non-integer value) edf represent an intermediate degree of smoothness (Wood 2006).

Reindeer density at the district-level showed temporal trends in both areas, whereas winter precipitation only showed a significant temporal trend in the poor area (Fig. 1; Table S4.1). There were no significant temporal trends in winter temperature in any of the areas, but average winter temperature in the poor area was significantly higher, i.e. closer to zero, compared to average temperature in the good area (Fig. 1; Table S4.1). In the analysis of summer conditions there were no significant differences in average conditions, and no significant differences in average values between the areas (Fig. 1; Table S4.1). Population density increased over time in both areas and the poor area showed a higher density than the good area (Fig. 1; Table S4.1; see also Supplement S1 for similar trends in the number of females per owner). Consequently, based on the difference in temperature and precipitation during winter we categorized our study area into a good and a poor area.

LITERATURE CITED

Wood, S. N. 2006. Generalized additive models: an introduction with R. Chapman & Hall/CRC.

Wood, S. N. 2012. mgcv: GAMs with GCV/AIC/REML smoothness estimation and GAMMs by PQL. R package version 1.7-28.

Table S4.1. Generalized additive models (GAM) showing how: (a) winter temperature; (b) winter precipitation; (c) summer temperature; (d) summer precipitation; and (e) population density are modeled as a smooth relationship of year separated by area (see Figure 1 for details). Estimated degrees of freedom (edf), which provide an estimate of the degree of complexity in the relationship, and deviance explained by the model (D) are also provided.

Parametric coefficients					Smooth terms			
Parameter	Value	St. err	*t*	*P*	Parameter	edf	*F*	*P*
(a) Winter temperature, °C (D = 92.7%, *n* = 24)								
Intercept	-5.026	0.279	-18.040	<0.001	s(year) × Area [Poor]	1.425	0.394	0.643
Area [Good]	-6.099	0.394	-15.480	<0.001	s(year) × Area [Good]	1.390	0.525	0.563
(b) Winter preciptation, mm (D = 87.1%, *n* = 24)								
Intercept	3.692	0.193	19.150	<0.001	s(year) × Area [Poor]	1.874	7.561	0.004
Area [Good]	-2.908	0.273	-10.670	<0.001	s(year) × Area [Good]	1.000	0.433	0.519
(c) Summer temperature, °C (D = 1.9%, *n* = 24)								
Intercept	8.881	0.228	38.905	<0.001	s(year) × Area [Poor]	1.000	0.000	0.988
Area [Good]	-0.167	0.323	-0.518	0.610	s(year) × Area [Good]	1.000	0.120	0.733
(d) Summer preciptation, mm (D = 11.5%, *n* = 24)								
Intercept	3.514	0.267	13.187	0.610	s(year) × Area [Poor]	1.000	1.349	0.259
Area [Good]	0.130	0.377	0.344	0.734	s(year) × Area [Good]	1.000	1.131	0.300
(e) Population density, km^{-2} (D = 99.5%, *n* = 24)								
Intercept	0.398	0.015	27.230	<0.001	s(year) × Area [Poor]	1.837	56.820	<0.001
Area [Good]	1.222	0.021	59.170	<0.001	s(year) × Area [Good]	1.950	52.540	<0.001
